# Engineered small metal‐binding protein tag improves the production of recombinant human growth hormone in the periplasm of *Escherichia coli*

**DOI:** 10.1002/2211-5463.12808

**Published:** 2020-03-09

**Authors:** David A. Perez‐Perez, Elizeth Pioquinto‐Avila, Eder Arredondo‐Espinoza, Jose Ruben Morones‐Ramirez, Isaias Balderas‐Renteria, Xristo Zarate

**Affiliations:** ^1^ Universidad Autonoma de Nuevo Leon Facultad de Ciencias Quimicas San Nicolas de los Garza Mexico; ^2^ Universidad Autonoma de Nuevo Leon Facultad de Ciencias Quimicas Centro de Investigacion en Biotecnologia y Nanotecnologia Parque de Investigacion e Innovacion Tecnologica Apodaca Mexico

**Keywords:** *Escherichia coli*, human growth hormone, PelB‐SmbP, periplasm, protein expression and purification, SmbP

## Abstract

Fusion proteins play an important role in the production of recombinant proteins in *Escherichia coli.* They are mostly used for cytoplasmic expression since they can be designed to increase the solubility of the target protein, which then can be easily purified via affinity chromatography. In contrast, fusion proteins are not usually included in construct designs for periplasmic production. Instead, a signal sequence is inserted for protein transport into the periplasm and a C‐terminal his‐tag added for subsequent purification. Our research group has proposed the small metal‐binding protein (SmbP) isolated from the periplasm of *Nitrosomonas europaea* as a new fusion protein to express recombinant proteins in the cytoplasm or periplasm of *E. coli*. SmbP also allows purification via immobilized metal affinity chromatography using Ni(II) ions. Recently, we have optimized the periplasmic production of proteins tagged with SmbP by exchanging its native signal peptide with one taken from pectate lyase B (PelB), substantially increasing the amount of protein produced. In this work, we have expressed and purified soluble bioactive human growth hormone (hGH) tagged with PelB‐SmbP and obtained the highest periplasmic production reported for this protein so far. Its activity, tested on Nb2‐11 cells, was equivalent to commercial growth hormone at 50 ng·mL^−1^. Therefore, we strongly recommend the use of PelB‐SmbP as a protein tag for the expression and purification of hGH or other possible target proteins in the periplasm of *E. coli.*

## Graphical abstract

This work describes the production of bioactive human growth hormone tagged with the fusion protein PelB‐SmbP in the periplasm of *Escherichia coli.* Here, we report the highest periplasmic production for this protein so far. Therefore, PelB‐SmbP is an attractive tag for the expression and purification of other target therapeutic proteins in *Escherichia coli.*

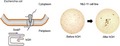

**Abbreviations**
dNTP: deoxynucleotide triphosphate
hGH: human growth hormone
IMAC: immobilized metal affinity chromatography
LB: Luria–Bertani broth
OD_600_: optical density at 600 nm
PelB: pectate lyase B
SmbP: small metal‐binding protein
Tris: Tris(hydroxymethyl)‐aminomethane

Many biopharmaceuticals are currently produced recombinantly in different microorganisms 1. *Escherichia coli* is preferred because it can produce large quantities at low cost 2. Unfortunately, *E. coli* has significant disadvantages as a host organism since it cannot carry out certain post‐translational modifications, and often, the protein of interest can form inclusion bodies. Fusion proteins have helped greatly to deal with this problem since they can be designed to function as solubility tags, and some also allow purification via affinity chromatography 3. Recombinant proteins produced in *E. coli* can be accumulated in the cytoplasm or periplasm 4; usually, the cytoplasm is the first choice due to its higher protein production, but a drawback can be that it lacks the proper environment to facilitate disulfide bond formation. Within the periplasm, the Dsb protein family assists with proper folding for these proteins 5. Periplasmic expression offers additional advantages; for example, it contains much lower amounts of proteins and nucleic acids that can interfere with the purification process, and the periplasmic fraction can be easily obtained via an osmotic shock 6, 7.

Recently, we have proposed two new fusion proteins: SmbP from *Nitrosomonas europaea* and CusF from *E. coli* 8, 9, 10. The biology of both proteins involves the removal of metal ions from the bacteria, SmbP binding Ni(II), Cu(II), Zn(II), and Fe(III) ions 11, whereas CusF can bind Cu(I), Ag(I), and Cu(II) 9. Both proteins are naturally found in the cell periplasm, and they contain a signal peptide dependent on the Sec pathway for transport to this location. Experiments in our laboratory have shown that SmbP and CusF function as solubility tags and can be purified employing IMAC, obtaining high purity, and very importantly, excellent final yields since these proteins have a molecular weight of only 10 kDa. We have made improvements to both proteins to increase protein quantity and purity 12, 13. In the case of SmbP, its signal peptide was exchanged for three different sequences: the signal peptides from the proteins CusF, PelB (*Erwinia carotovora*), and TorA (*E. coli*)*.* The first two signal transport to occur via the Sec route and the third via the Tat route. Using any of the three different signal peptides, the periplasmic expression of fluorescent proteins marked with SmbP increased more than a 1000‐fold, PelB‐SmbP being slightly better than CusF‐SmbP. As for TorA‐SmbP, it transported folded proteins to the cell periplasm with a little less efficiency; although protein production with any of the different signal sequences is target protein‐dependent 13.

The human growth hormone (hGH) is a protein produced, stored, and secreted by the anterior pituitary gland. It maintains positive nitrogen balance and initiates protein synthesis in muscle cells, raises amino acid uptake into skeletal muscle, regulates longitudinal bone growth, and protects cardiac myocytes and lymphoid cells against apoptosis 14. Due to its variety of biological roles, hGH has been approved for the treatment of the following conditions: GH deficiency, idiopathic short stature, chronic kidney disease, Turner syndrome, Prader–Willi syndrome, SHOX gene haploinsufficiency, Noonan syndrome, and small for gestational age infants 15. The incidence in children with hGH deficiency has been estimated to be about 1 in 4000 16.

Mature hGH consists of 191 amino acid residues, with four cysteines being involved in two disulfide bonds 17. Therefore, this protein is appropriate for expression in the periplasm of *E. coli* and for tagging with PelB‐SmbP. In this work, we describe the expression and purification of recombinant hGH tagged with PelB‐SmbP. Purification was carried out with an osmotic shock followed by metal affinity chromatography, and finally, the SmbP tag was removed with enterokinase. Pure hGH was assayed on the Nb2‐11 cell line showing activity similar to that of commercial serotropin as positive control.

## Materials and methods

### DNA constructs

The pET30a vector containing the PelB‐SmbP sequence (cloned using the NdeI and KpnI restriction sites) 13 was digested with NcoI and XhoI. The DNA sequence for hGH was optimized for *E. coli* expression and synthesized by GenScript (Piscataway, NJ, USA). After digestion with the same enzymes, it was ligated into pET30a‐PelB‐SmbP. The construct contains an enterokinase recognition sequence for tag removal, located between the fusion protein and hGH. *E. coli* strain DH5α was used for general cloning procedures and DNA maintenance. All constructs used in this work were fully characterized by sequencing.

### Protein expression

The expression plasmid pET30a‐PelB‐SmbP‐hGH was transformed into *E. coli* BL21(DE3) cells. For small‐scale expression experiments, a single colony was used to inoculate LB broth–kanamycin (30 µg·mL^−1^) and incubated at 37 °C. Once the OD_600_ reached 0.4–0.6, protein expression was induced by the addition of 1 m IPTG to a final concentration of 0.1 mm. The culture was incubated for 16 h at 25 °C. For 1‐L expression, overnight cultures were used to inoculate LB–kanamycin in baffled flasks and incubated at 37 °C until OD_600_ reached 0.4–0.6. IPTG was added to a final concentration of 0.1 mm, and again, the culture was incubated for 16 h at 25 °C.

### Protein purification

Cells from the 1‐L culture were harvested by centrifugation at 4 °C, and SmbP‐hGH was extracted from the periplasm by osmotic shock 13. The cells were resuspended in a hypertonic solution (200 mm Tris/HCl, 20% sucrose, 2 mm EDTA, 0.5 mg·mL^−1^ lysozyme, pH 8.0) and incubated for 1 h at 4 °C. Cells were centrifuged at 24 500 ***g*** for 10 min and the supernatant collected. The remaining pellet was resuspended in water and incubated for 1 h at 4 °C followed by centrifugation at 24 500 ***g*** for 10 min at 4 °C. The supernatant from the hypotonic solution was combined with the supernatant from the hypertonic solution and dialyzed against buffer A (50 mm Tris/HCl, 500 mm NaCl, pH 8.0) at 4 °C. After dialysis, the supernatant was clarified by centrifugation at 24 500 ***g*** for 10 min. Purification was performed using the ÄKTA Prime Plus FPLC (GE Healthcare, Chicago, IL, USA). The protein solution was loaded into a 5 mL HisTrap FF previously charged with Ni(II) and equilibrated with buffer A. The column was washed with buffer A until no more absorption at 254 nm was observed. hGH was eluted with an imidazole gradient (up to 200 mm) in 40 column volumes. All eluted fractions were analyzed by SDS/PAGE; the purity of hGH was calculated by densitometry using the software imagej (http://imagej.nih.gov/ij). Protein content was quantified by the Bradford reagent using BSA as the standard. One milligram of SmbP‐hGH was digested with 50 units of enterokinase light chain (New England Biolabs, Ipswich, MA, USA) in a 2 mL reaction volume for 16 h at room temperature. After cleavage, the protein solution was dialyzed against 700 mL of buffer A for 1 h (this step was performed three times, the last for 16 h) and incubated with Profinity IMAC resin (Bio‐Rad Laboratories, Hercules, CA, USA) charged with Ni(II) for 1 h. The resin was then centrifuged at 1500 ***g*** for 30 s, and the supernatant was collected and analyzed by SDS/PAGE as our final product.

### *In vitro* cell proliferation assay

The biological activity of hGH was tested using Nb2‐11 cells. The cells were cultured in suspension in Fischer’s medium supplemented with 10% fetal bovine serum, 10% horse serum, 0.075% sodium bicarbonate, 0.05 mm β‐mercaptoethanol, and 2 mm glutamine at 37 °C in a humidified atmosphere containing 5% CO_2_. The cells were harvested by centrifugation and resuspended in the same medium without fetal bovine serum for 24 h to slow the rate of cell division. For the activity assay, approximately 2 × 10^4^ cells were seeded into a 96‐well microtiter plate containing 100 µL of assay medium. Proliferation was promoted by adding different amounts of our purified hGH or a commercial product (serotropin from PiSA Pharmaceuticals, Guadalajara, JC, Mexico) over a concentration range of 3–50 ng·mL^−1^. Cell growth was evaluated using the WST‐1 assay 18, after 24 h of incubation with hGH.

### Statistical analysis

The statistical analysis was done using graphpad prism version 5.0 (San Diego, CA, USA). Experiments were performed in triplicate. One‐way ANOVA was used for multiple group comparisons (*P < 0.05). Tukey’s test was used to calculate statistical significance.

## Results and Discussion

**Fig. 1:**
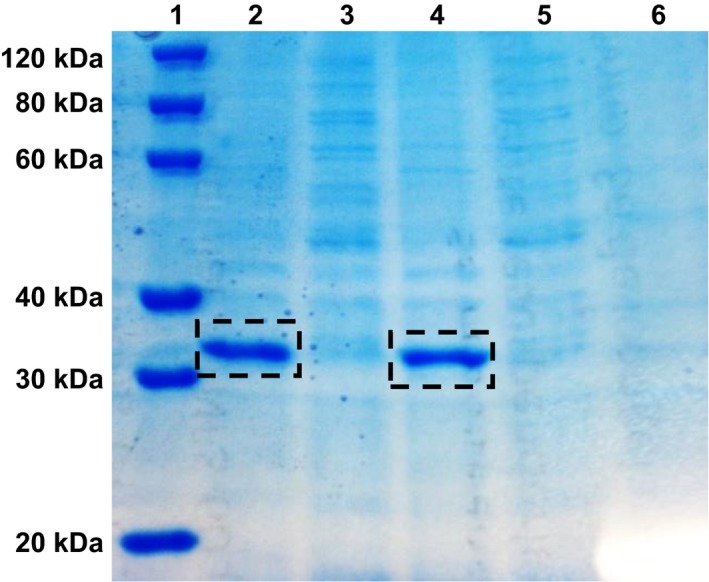
12% SDS/PAGE analysis of two small‐scale periplasmic expression of PelB‐SmbP‐hGH. Lane 1: protein marker; lanes 2 and 4: hypotonic fractions; lanes 3 and 5: hypertonic fractions; lane 6: hypotonic fraction from uninduced cells. Calculated molecular weight for SmbP‐hGH: 32 kDa.

**Fig. 2:**
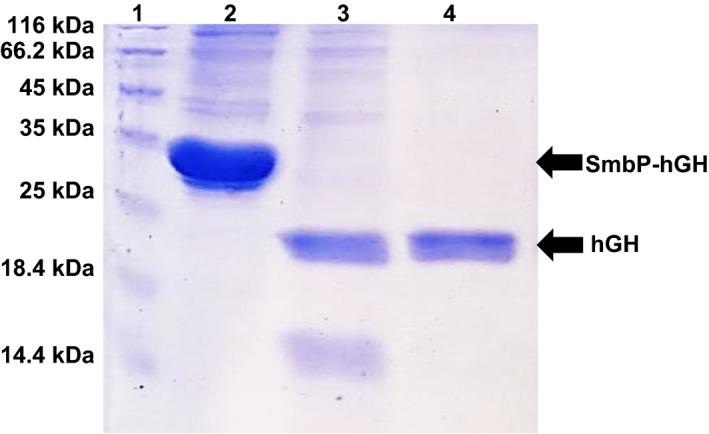
15% SDS/PAGE analysis of hGH after final purification and SmbP tag removal. Lane 1: protein marker; lane 2: SmbP‐hGH after IMAC purification; lane 3: SmbP‐hGH after reaction with enterokinase; lane 4: pure hGH after second IMAC purification to remove SmbP. Calculated molecular weight for hGH: 22 kDa.

In order to first verify whether SmbP‐hGH was being produced in the periplasm, we performed small‐scale expression experiments followed by an osmotic shock. Figure 1 shows the SDS/PAGE analysis of the hypertonic and hypotonic fractions. A protein band, corresponding to the expected position of SmbP‐hGH (approximately 32 kDa), was observed only in the hypotonic fractions. Previous experiments performed in our laboratory showed that red fluorescent protein tagged with PelB‐SmbP was mostly present in that same fraction 13. Interestingly, when the process was upscaled to a larger volume, some SmbP‐hGH was also observed in the hypertonic fraction. For this reason, both fractions were pooled and dialyzed for protein purification. Figure S1 shows the SDS/PAGE analysis from the IMAC purification and all elution fractions. After affinity chromatography, reaction with enterokinase, and a second chromatographic step to remove the SmbP tag, 98% pure recombinant hGH was obtained, a single band with a molecular mass of 22 kDa (Fig. 2). Therefore, the total quantity of hGH present in one liter of cell culture is 15.5 mg. Table 1 shows the full summary purification table 19, including percentage yields.

**Table 1:** Purification summary for hGH tagged with PelB‐SmbP from the periplasmic fraction of 1 L of cell culture

Purification step	Volume (mL)	Pellet weight (g)	Total protein (mg)	Amount of hGH (mg)	Yield (%)	Purity (%)
Bacterial culture	1000	5.4	–	–	–	–
Crude extract	100	–	546	65.5	100	12
IMAC	40	–	24.1	21.7	33.1	90
Cleavage and IMAC	40	–	15.8	15.5	23.6	98

Different methodologies have been used to produce hGH in *E. coli.* Some of these include working with inclusion bodies and three or more purification steps, resulting in low yields or high costs 20. One report for the production of hGH in the periplasm of *E. coli* showed a yield of 1.4 mg·L^−1^ culture of purified hGH, using PelB as a signal peptide and a C‐terminal his‐tag 21. Our work clearly indicates that SmbP vastly improved hGH expression (10‐fold), as compared with the his‐tag alone.

**Fig. 3:**
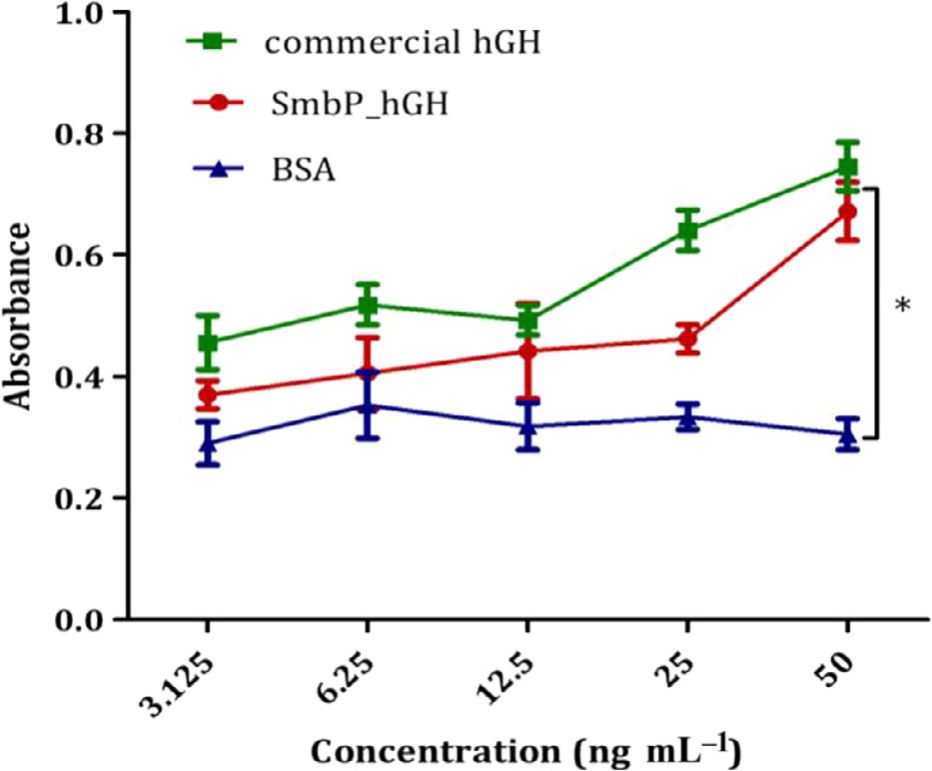
Cell proliferation assay of purified hGH in the Nb2‐11 cell line. Dose–response proliferation curve of Nb2‐11 cells by exposure to different concentrations of purified hGH, commercial hGH, and BSA as a control. Error bars represent standard deviation from three independent experiments, *n* = 3, and *P < 0.05. Table S1 shows the complete ANOVA and Tukey’s tests.

In terms of the bioactivity of the recombinant hGH, Fig. 3 shows the results of a cell proliferation assay using the Nb2‐11 cell line. This line derives from a transplantable lymphoma from the lymph nodes of a male rat of the Noble strain. Following prolonged estrogen treatment, cellular proliferation is dependent on mammalian lactogens. This feature allows this cell line to be used in the determination of the biological activity of hGH 22. Cells in the assay medium were treated with purified or commercial hGH, and BSA (as a negative control). Both hGH proteins show the ability to induce cell proliferation in a dose‐dependent manner up to 50 ng·mL^−1^, as previously reported 17, 23; no cell proliferation was observed with BSA. These results demonstrate that hGH tagged with PelB‐SmbP and purified with our methodology has biological activity.

## Conclusions

We report for the first time the use of SmbP for the production of a biopharmaceutical product, describing an efficient and straightforward strategy for the production of hGH tagged with PelB‐SmbP in the periplasm of *E. coli*. After the cleavage of SmbP, we have been able to produce 15.5 mg of active hGH at 98% purity per liter of culture. This technique could be useful for the production of other therapeutic proteins.

## Conflict of interest

The authors declare no conflict of interest.

## Author contributions

DAP‐P made the DNA constructs, expressed and purified the recombinant human growth hormone, performed cell culture assays, and wrote the manuscript. EP‐A and EA‐E performed cell culture assays. JRM‐R and IB‐R assisted with data interpretation and edited the manuscript. XZ designed the experiments, edited the manuscript, and proposed the research as principal investigator. All authors read and approved the final manuscript.

## Supporting information


**Fig. S1.** 12% SDS/PAGE analysis of SmbP_hGH after first IMAC purification. Lane 1: protein marker; Lane 2: periplasmic hypotonic fraction; Lane 3: periplasmic hypotonic fraction; Lane 4: flow‐through; Lane 5‐51 elution fractions.
**Fig. S2.** Cell proliferation assay of purified hGH in the Nb2‐11 cell line. Nb2‐11 cells were exposure to 50 ng/ ml of purified hGH, commercial hGH, and bovine serum albumin as a control. Here, polyhistidine tagged enterokinase was used to cleave SmbP from hGH, thus, after second IMAC purification, the enterokinase enzyme was completely removed.
**Table S1.** Statistical analysis of the cell proliferation assay. Turkey’s test was used to calculate statistical significance. One‐way ANOVA was used for multiple group comparisons (*p < 0.05).
