# Supplementary material for: Engineered small metal‐binding protein tag improves the production of recombinant human growth hormone in the periplasm of Escherichia coli
Source: FEBS Open Bio. 2020 Mar 9;10(4):546–51. doi: 10.1002/2211-5463.12808 (PMC7137794; doi:10.1002/2211-5463.12808)
Supplement: Supplementary file 1 — Fig. S1. 12% SDS/PAGE analysis of SmbP_hGH after first IMAC purification. Lane 1: protein marker; Lane 2: periplasmic hypotonic fraction; Lane 3: periplasmic hypotonic fraction; Lane 4: flow‐through; Lane 5‐51 elution fractions. Fig. S2. Cell proliferation assay of purified hGH in the Nb2‐11 cell line. Nb2‐11 cells were exposure to 50 ng/ ml of purified hGH, commercial hGH, and bovine serum albumin as a control. Here, polyhistidine tagged enterokinase was used to cleave SmbP from hGH, thus, after second IMAC purification, the enterokinase enzyme was completely removed. Table S1. Statistical analysis of the cell proliferation assay. Turkey’s test was used to calculate statistical significance. One‐way ANOVA was used for multiple group comparisons (*p < 0.05). [file FEB4-10-546-s001.pptx]

## Slide 1
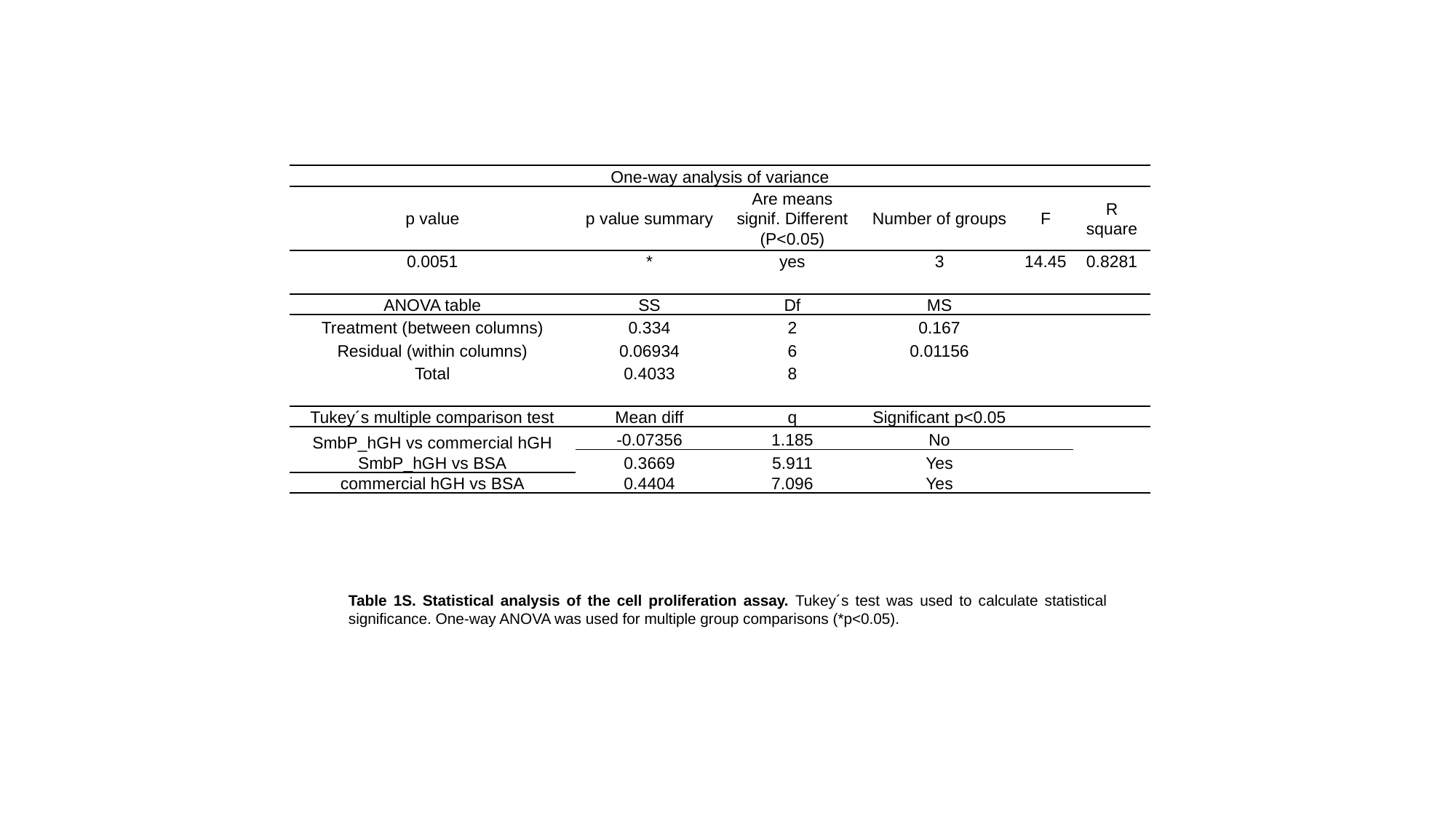

| One-way analysis of variance | | | | | |
| --- | --- | --- | --- | --- | --- |
| p value | p value summary | Are means signif. Different (P<0.05) | Number of groups | F | R square |
| 0.0051 | \* | yes | 3 | 14.45 | 0.8281 |
| | | | | | |
| ANOVA table | SS | Df | MS | | |
| Treatment (between columns) | 0.334 | 2 | 0.167 | | |
| Residual (within columns) | 0.06934 | 6 | 0.01156 | | |
| Total | 0.4033 | 8 | | | |
| | | | | | |
| Tukey´s multiple comparison test | Mean diff | q | Significant p<0.05 | | |
| SmbP\_hGH vs commercial hGH SmbP\_hGH vs BSA | -0.07356 | 1.185 | No | | |
| | 0.3669 | 5.911 | Yes | | |
| commercial hGH vs BSA | 0.4404 | 7.096 | Yes | | |
Table 1S. Statistical analysis of the cell proliferation assay. Tukey´s test was used to calculate statistical significance. One-way ANOVA was used for multiple group comparisons (*p<0.05).

## Slide 2
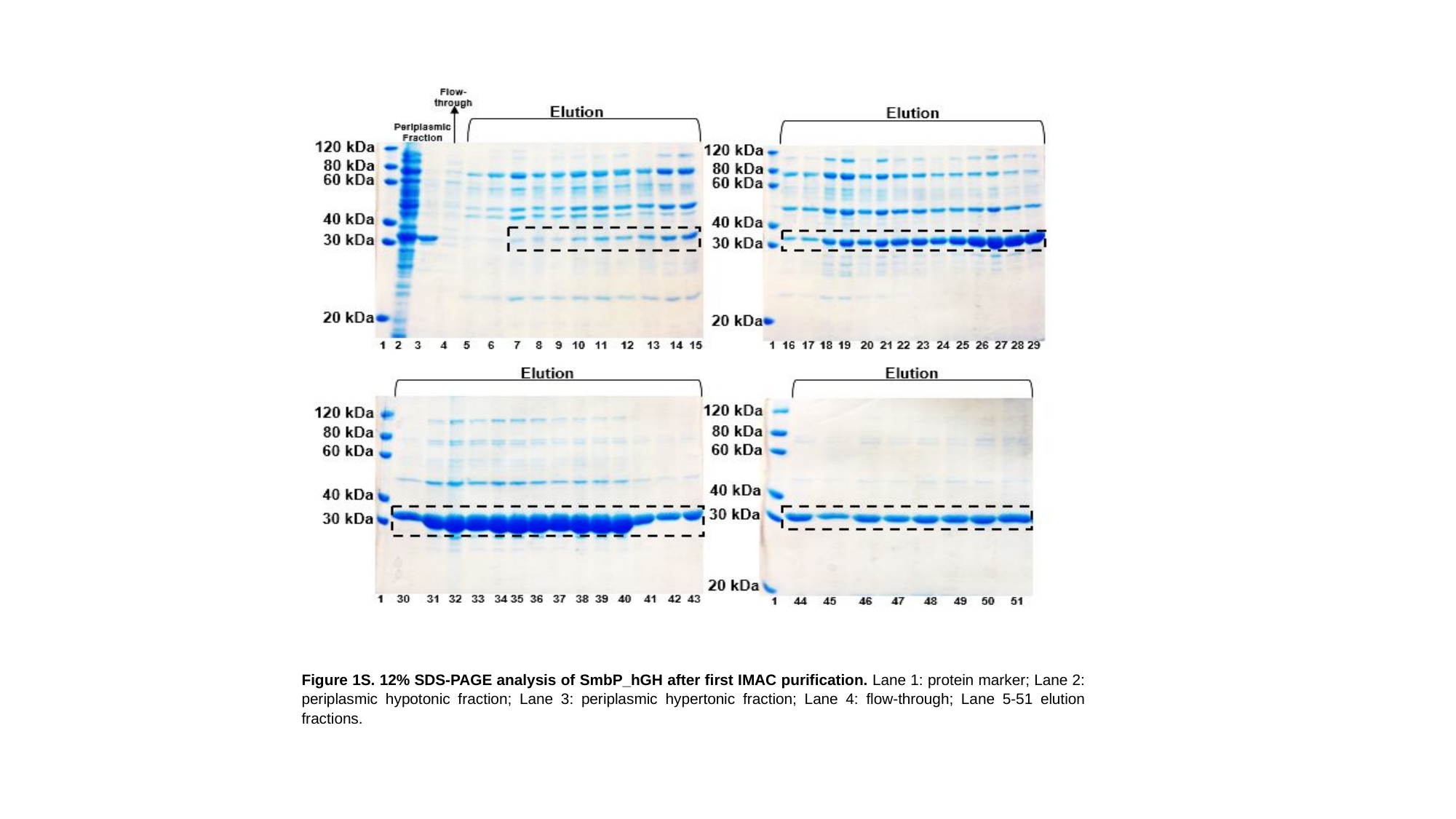

Figure 1S. 12% SDS-PAGE analysis of SmbP_hGH after first IMAC purification. Lane 1: protein marker; Lane 2: periplasmic hypotonic fraction; Lane 3: periplasmic hypertonic fraction; Lane 4: flow-through; Lane 5-51 elution fractions.

## Slide 3
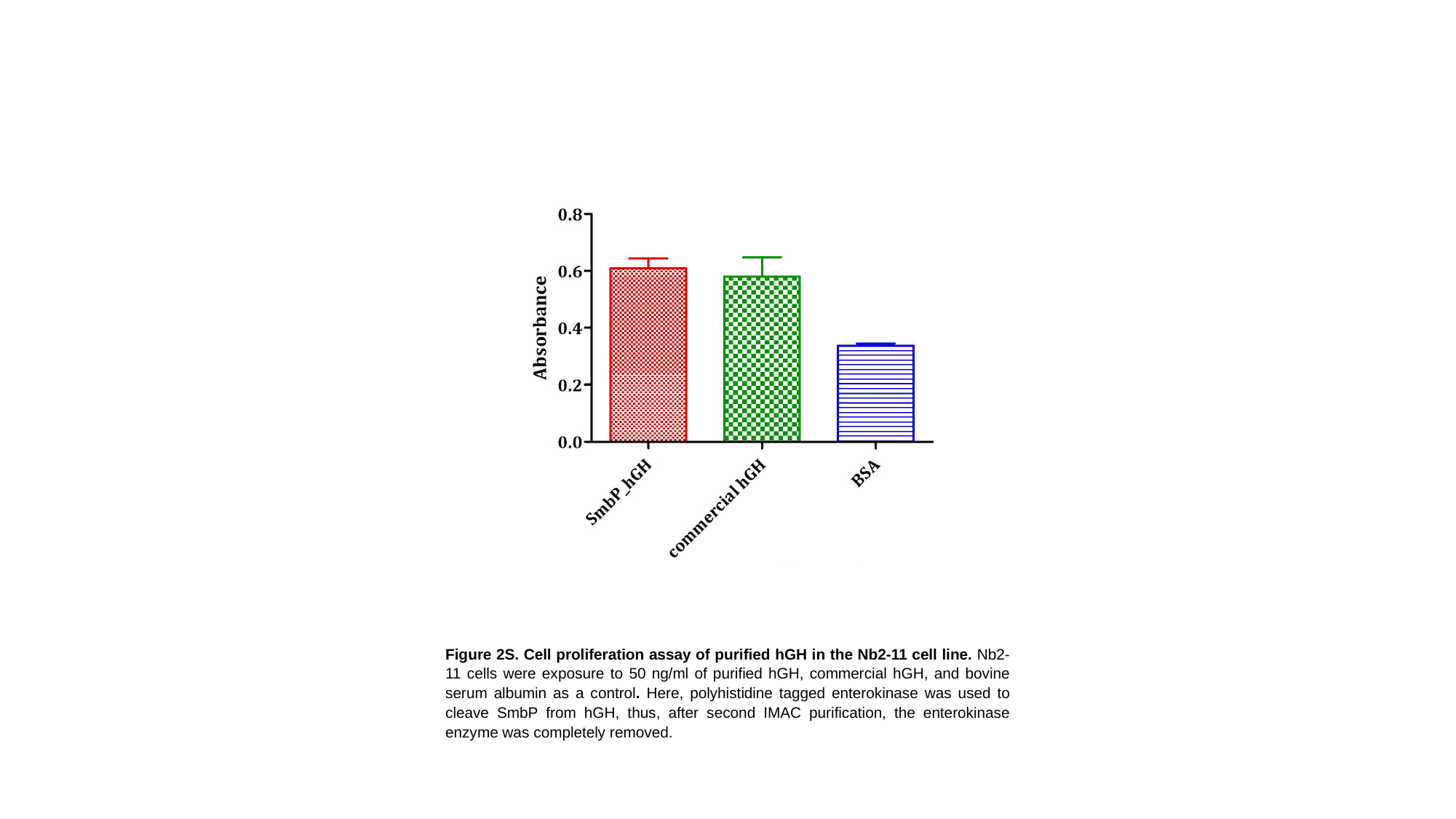

Figure 2S. Cell proliferation assay of purified hGH in the Nb2-11 cell line. Nb2-11 cells were exposure to 50 ng/ml of purified hGH, commercial hGH, and bovine serum albumin as a control. Here, polyhistidine tagged enterokinase was used to cleave SmbP from hGH, thus, after second IMAC purification, the enterokinase enzyme was completely removed.
